# Effects of Botulinum Toxin Injection on Masticatory Muscle Activity in Sleep Bruxism: A Systematic Review and Meta-Analysis

**DOI:** 10.3390/jcm15187179

**Published:** 2026-09-16

**Authors:** Matteo Val, Anna Colonna, Laura Nykänen, Matteo Pollis

**Affiliations:** 1 Department of Medical Biotechnology, School of Dentistry, University of Siena, 53100 Siena, Italymatteo.pollis@gmail.com (M.P.); 2 Unit of Oral and Maxillofacial Surgery, Ca’ Foncello Hospital, AULSS2 Marca Trevigiana, 31100 Treviso, Italy; 3 Department of Oral and Maxillofacial Diseases, University of Helsinki, 00100 Helsinki, Finland; lauraolivianykanen@gmail.com; 4 Clinic of Oral and Maxillofacial Diseases, Head and Neck Center, Helsinki University Hospital, 00029 Helsinki, Finland

**Keywords:** bruxism, botulinum toxin, sleep bruxism, awake bruxism, electromyography, systematic review, meta-analysis

## Abstract

**Background/Objectives:** Bruxism, encompassing both awake (AB) and sleep bruxism (SB), is a prevalent condition associated with involuntary jaw muscle activity, leading to tooth wear and muscle hypertrophy. Botulinum toxin type A (BoNT-A) has emerged as a potential treatment by reducing masticatory muscle hyperactivity. This systematic review aimed to evaluate the efficacy of administering BoNT-A injections into the masticatory muscles for reducing bruxism-related muscle activity, as measured by any validated instrumental or questionnaire-based method. **Methods:** A systematic search of PubMed, Scopus, and Web of Science was performed according to PRISMA guidelines, with the search updated through July 2026. Eight studies evaluating bruxism/masticatory muscle activity after BoNT-A injection, using electromyography (EMG), polysomnography, or validated questionnaires/clinical global impression scales, met the inclusion criteria. Five placebo-controlled randomized controlled trials were synthesized quantitatively; a meta-analysis using a random-effects (DerSimonian–Laird) model, chosen a priori given the anticipated methodological heterogeneity across studies and confirmed by a fixed-effect model computed as a sensitivity analysis, was used to calculate the pooled Hedges’ g standardized mean differences with 95% confidence intervals (CIs) for bruxism/muscle-activity outcomes. Three additional studies with non-placebo designs—two active-comparator RCTs and one large observational study—were synthesized narratively because their designs (no placebo arm) were not compatible with pooling. Risk of bias was assessed with the Cochrane Risk of Bias 2.0 tool and the Newcastle–Ottawa Scale. **Results:** Because the five placebo-controlled trials captured two related but distinct constructs of muscle activity, construct-specific estimates are presented as the primary quantitative findings: a fixed-effect pooled Hedges’ g of −0.70 (95% CI: −1.04 to −0.37) for studies measuring bruxism event frequency, and −1.31 (95% CI: −1.95 to −0.68) for studies measuring EMG signal amplitude, in patients with sleep bruxism (SB) compared with placebo. As a secondary, exploratory summary across both constructs, the overall random-effects pooled estimate was Hedges’ g = −1.09 (95% CI: −1.65 to −0.54), with moderate-to-substantial heterogeneity (I^2^ = 58.6%). The formal test for the numerical difference between these two construct-specific estimates was not statistically significant (*p* = 0.096), reflecting the small number of studies per subgroup; the overall pooled estimate is therefore best interpreted as a secondary, composite signal across related constructs. The three non-pooled studies—two active-comparator RCTs and a large real-world observational series (number of partecipants = 304)—independently reported reductions in masticatory muscle EMG amplitude after BoNT-A injection, consistent in direction with the pooled estimate. In contrast, of the six studies that also assessed bruxism frequency by questionnaire, clinical global impression, or PSG scoring, only one found a clear BoNT-A-specific benefit; the remaining five found no significant difference from the comparator, even when an EMG-based measure from the same participants did show a BoNT-A-specific reduction. Mild and transient adverse effects were reported, with no serious events. The certainty of evidence was rated as low-to-moderate regarding the precise magnitude of EMG-based muscle activity reduction (downgraded for construct heterogeneity and imprecision) and very low regarding any corresponding reduction in questionnaire- or clinically-assessed bruxism frequency, and low concerning the short-term safety of BoNT-A administration (downgraded from the RCT-default starting point for imprecision, given the small number of controlled participants and short follow-up, and for indirectness, given heterogeneous dosing protocols across studies). **Conclusions:** BoNT-A injections into the masticatory muscles consistently reduce EMG-detectable masticatory muscle activity in patients with SB, although the magnitude of this effect varies depending on whether event frequency or signal amplitude is used to quantify it. This EMG-measured reduction did not consistently correspond to a reduction in bruxism frequency as assessed by questionnaires, clinical global impression, or PSG scoring, and the two types of measures should not be assumed interchangeable when interpreting treatment response. BoNT-A consistently reduces EMG-detectable masticatory muscle activity and, on this mechanistic basis, may be considered a candidate adjunctive therapy, particularly for individuals unresponsive to conservative treatments; claims of clinical efficacy should specify which type of bruxism measure they refer to, since this review does not provide direct evidence of benefit on pain, function, or other bruxism-related symptoms. Future research should focus on standardizing injection protocols and outcome measures—ideally reporting instrumental and questionnaire-based outcomes concurrently—and on evaluating long-term safety and efficacy.

## 1. Introduction

Bruxism is a repetitive masticatory muscle activity characterized by clenching or grinding of the teeth and/or by bracing or thrusting of the mandible [1,2]. It is commonly divided into two distinct forms: awake bruxism (AB), often associated with psychosocial stress and teeth contact, clenching, or bracing, and sleep bruxism (SB), considered a sleep-related movement disorder with involuntary masticatory muscle activity, namely clenching, grinding, or bracing during sleep [3]. Both phenotypes are thought to be centrally regulated [4,5], and a valid assessment requires a graded diagnostic approach, distinguishing “possible” bruxism (based on self-report alone), “probable” bruxism (self-report plus positive clinical inspection), and “definite” bruxism (instrumental confirmation, e.g., electromyography (EMG) or polysomnography (PSG), with or without self-report and clinical findings) [1].

Bruxism is prevalent in both adults [6] and the young population [7,8], with varying degrees of clinical impact. Complications include dental wear [9], tooth fractures, prosthetic damage [10,11], temporomandibular disorders (TMD) [12], headache [13], and orofacial pain [14]. These consequences can lead to significant deterioration in oral health-related quality of life [8].

Conceptually, the management of bruxism can pursue two distinct goals: alleviating the negative consequences of bruxism (e.g., pain, tooth wear) or reducing the underlying masticatory muscle overactivity that is hypothesized to drive those consequences [15]. Current conservative approaches for the negative consequences of bruxism include behavioral modifications, physiotherapy, pharmacological treatments, and occlusal appliances [15]; however, especially for SB, these strategies do not directly target muscle hyperactivity, and conventional therapies often fail to fully control it or to prevent progression of dental damage. Reducing muscle activity itself, consistent with the overload theory, can in principle be pursued at the central level (e.g., stress management, pharmacotherapy) or at the peripheral level, by directly and reversibly modulating neuromuscular transmission.

Botulinum toxin (BoNT), a neurotoxin derived from the bacterium *Clostridium botulinum*, acts by inhibiting the release of acetylcholine at neuromuscular junctions, leading to a temporary and reversible reduction in muscle activity [16,17,18,19]. This peripheral, dose-dependent paralyzing action has made BoNT a candidate tool for directly targeting overactive masticatory muscles in bruxism, and several studies have investigated its effect on masticatory muscle activity in both AB and SB, with promising—yet heterogeneous—results [20,21,22,23,24,25,26,27,28,29,30,31]. Beyond bruxism management, BoNT-A injections into the masticatory muscles have also been explored as an adjunct in orthognathic surgery and maxillofacial trauma management, further underscoring the breadth of clinical applications targeting masticatory muscle activity [32].

EMG remains the most widely used instrumental measure of masticatory muscle activity in this population; however, it currently lacks universally accepted diagnostic cut-off values, which limits its use as a stand-alone diagnostic criterion even though it is well suited for quantifying within-subject change after an intervention [1]. Because muscle activity and clinical symptoms (e.g., pain, functional limitation) are conceptually distinct constructs that do not necessarily change in parallel, mixing them within a single set of outcomes can obscure whether BoNT-A achieves its proposed mechanistic target. The present review therefore restricts its scope to the mechanistic question of muscle activity, deliberately outside the scope of pain or other bruxism-related symptoms, which warrant dedicated investigation.

Accordingly, the objective of this systematic review is to evaluate the available randomized controlled trial and observational evidence on the effect of BoNT-A injections into the masticatory muscles on EMG-measured muscle activity in adults with AB and SB, and to analyze outcome variability based on methodological differences across studies; as detailed in Section 3.3, the eligible evidence base identified was almost exclusively derived from SB populations, and this asymmetry is reflected throughout the Results and Discussion.

## 2. Materials and Methods

### 2.1. Study Design and Registration

This systematic review followed the PRISMA 2020 guidelines [33] (see Supplementary Material S4 for the completed PRISMA 2020 checklist). A protocol was developed in accordance with PRISMA-P and registered on the PROSPERO database (ID: CRD420251037536).

### 2.2. PICO Framework

The review question was structured according to the PICO framework:

- **Population (P):** Adults (≥18 years) diagnosed with sleep or awake bruxism.
- **Intervention (I):** Injection of BoNT-A into masticatory muscles (masseter and/or temporalis or other masticatory muscles).
- **Comparison (C):** Placebo, no treatment, or other conservative management approaches.
- **Outcome (O):** Reduction in bruxism/masticatory muscle activity, operationalized according to whichever validated method each primary study used to quantify it: electromyographic (EMG) amplitude, EMG-derived bruxism events or Bruxism Index (BI), polysomnographically-scored rhythmic masticatory muscle activity (RMMA) parameters, or validated bruxism-frequency questionnaires/clinical global impression scales when used by a study as its measure of bruxism activity (not of pain). This deliberately broad, instrument-agnostic definition of the outcome reflects the substantial heterogeneity in bruxism assessment methodology across the field [1] and allows all eight included studies to contribute to the synthesis despite using different instruments. Because this instrument diversity has direct implications for how comparable the pooled effect sizes are, studies contributing to the quantitative synthesis were further classified post hoc by the general type of construct captured—event/episode frequency versus EMG signal amplitude/intensity—to allow a construct-based subgroup analysis (see Section 2.7). Adverse effects were recorded as a safety outcome. Pain, functional limitation, and other symptom-based or quality-of-life outcomes were not evaluated in this review, even when reported by a primary study alongside its muscle-activity data.

The PICO elements described above, extended with the study design and timeframe (PICOS), are summarized in Table 1.

### 2.3. Eligibility Criteria

Inclusion criteria:

- Human studies (randomized controlled trials or observational);
- Published between January 2005 and July 2026 (2005 was chosen as the lower bound because it approximates the period in which BoNT-A injections began to be systematically investigated for masticatory-muscle and TMD-related indications, following its earlier, more established therapeutic uses);
- Adults with a diagnosis of SB or AB;
- Use of BoNT-A as a therapeutic intervention;
- Reported a bruxism/masticatory muscle activity outcome measured by EMG (amplitude, events, or index), polysomnography, or a validated bruxism-frequency questionnaire/clinical global impression scale;
- Articles in English with full text available.

Exclusion criteria:

- Animal/in vitro studies;
- Case reports, narrative reviews, editorials;
- Studies using BoNT-A for indications unrelated to bruxism.

### 2.4. Search Strategy

**Figure 1:**
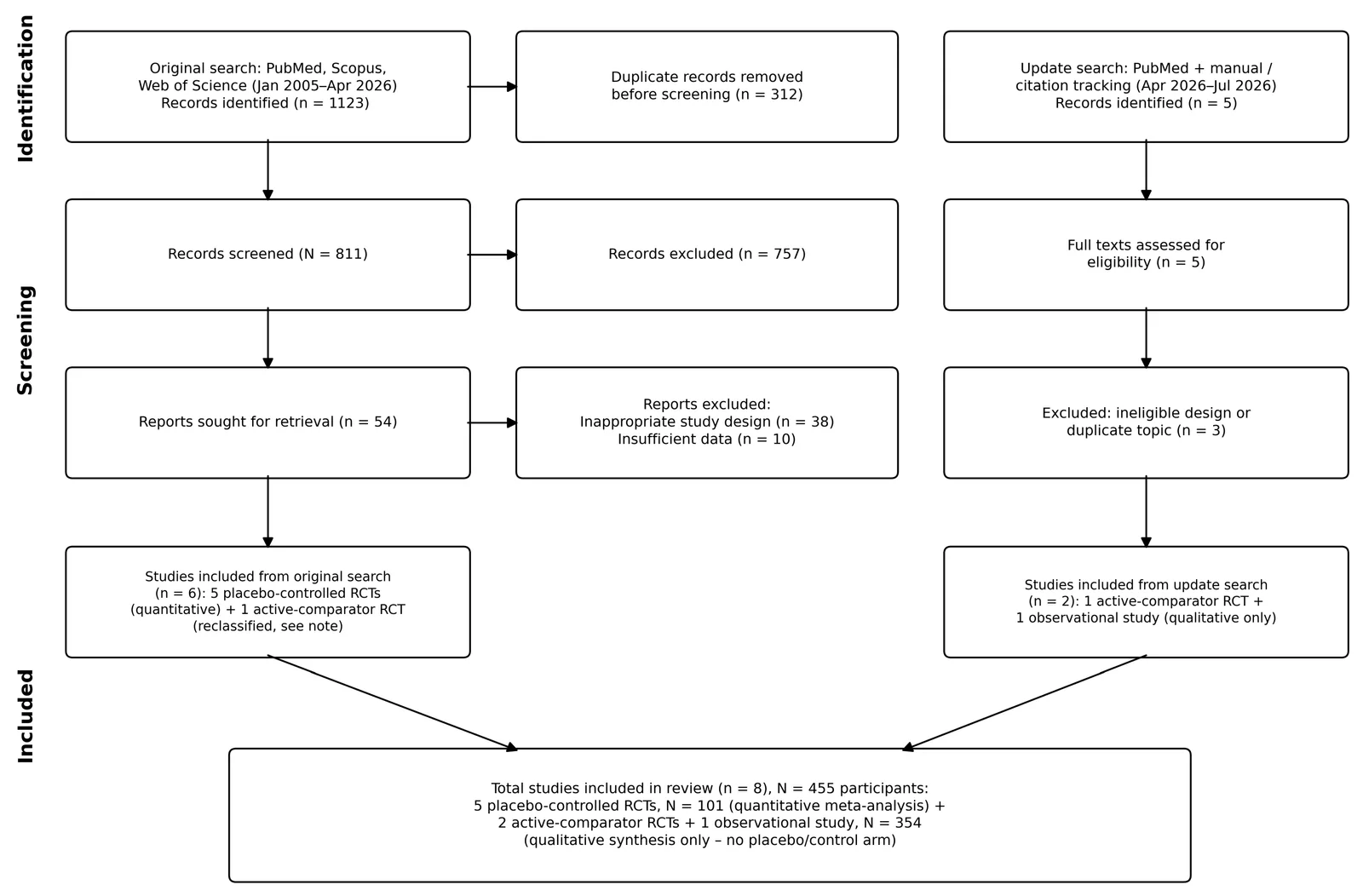
PRISMA flowchart of the study selection process.

Databases searched: PubMed, Scopus, and Web of Science, covering the literature through July 2026. Specifically, the original multi-database search (PubMed, Scopus, Web of Science) covered records from January 2005 to the 13th of April 2026 and was subsequently updated through the 27th of July 2026 using PubMed plus manual/citation tracking, as detailed in the PRISMA flow diagram (Figure 1). Keywords and MeSH terms included combinations of “bruxism,” “botulinum toxin,” “BoNT,” “masseter,” “temporalis,” “awake bruxism,” and “sleep bruxism,” supplemented by manual/citation tracking (see Figure 1). The Boolean combination of these terms, as the exact strings executed in each database, was as follows—PubMed: (“bruxism”[MeSH Terms] OR “bruxism”[All Fields] OR “sleep bruxism”[All Fields] OR “awake bruxism”[All Fields]) AND (“botulinum toxins”[MeSH Terms] OR “botulinum toxin”[All Fields] OR “BoNT”[All Fields] OR “botulinum toxin type A”[All Fields]) AND (“masseter”[All Fields] OR “temporalis”[All Fields] OR “masticatory muscle*”[All Fields]); Scopus: TITLE-ABS-KEY(bruxism OR “sleep bruxism” OR “awake bruxism”) AND TITLE-ABS-KEY(“botulinum toxin” OR BoNT) AND TITLE-ABS-KEY(masseter OR temporalis OR “masticatory muscle*”); Web of Science: TS=(bruxism OR “sleep bruxism” OR “awake bruxism”) AND TS=(“botulinum toxin” OR BoNT) AND TS=(masseter OR temporalis OR “masticatory muscle*”). Across the three databases, the original search retrieved 1123 records; after removal of 312 duplicates, 811 unique records were screened (see Figure 1 for the full PRISMA flow, including the update search). The original search of PubMed, Scopus, and Web of Science was conducted on 13 April 2026; PubMed was subsequently re-searched on 27 July 2026 as the update search to capture literature published since the original search date, consistent with the PRISMA flow diagram (Figure 1). Duplicate records were identified and removed using EndNote (version 20, Clarivate, Philadelphia, PA, USA) available to the authors at the time of the search.

### 2.5. Study Selection and Data Extraction

Three reviewers (M.V., A.C., M.P.) independently screened titles and abstracts. Full texts were assessed for eligibility. Discrepancies were resolved through discussion or by a fourth reviewer (L.N.).

Data extraction was performed independently and in duplicate by two reviewers (M.V. and M.P.), with disagreements resolved by discussion or by consulting a third reviewer (A.C.), consistent with the study selection process described above. A formal measure of inter-reviewer agreement (e.g., Cohen’s kappa) for title/abstract and full-text screening was not calculated; this is acknowledged as a limitation (see Section 4.2). Data extracted included: authors, publication year, country, study design, sample size, bruxism type, BoNT dose and site, comparator, follow-up period, and outcome measures—including all instruments each study used to assess bruxism activity (electromyography, polysomnography-derived indices, bruxism questionnaires, clinical global impression scales, or any other validated method), and for each instrumental (EMG/PSG-based) measure specifically, whether it captured event frequency or signal amplitude/intensity, to inform the construct-based analyses described under Data Synthesis—and adverse events.

### 2.6. Risk of Bias Assessment

- **RCTs:** Assessed using the Cochrane Risk of Bias 2.0 tool (randomization, allocation, blinding, incomplete outcome data, selective reporting) [34].
- **Observational studies:** Evaluated using the Newcastle–Ottawa Scale (selection, comparability, outcome) [35].

The certainty of the body of evidence for each outcome was assessed using the GRADE approach [36], considering risk of bias, inconsistency (including the construct heterogeneity described below), indirectness, imprecision, and publication bias.

### 2.7. Data Synthesis

Data were synthesized using both qualitative and quantitative methods. For the quantitative analysis, a meta-analysis was conducted to estimate the overall effect of BoNT-A on bruxism/muscle-activity outcomes across the five placebo-controlled RCTs (full computational details in the following paragraphs), using established meta-analytic formulae implemented in IBM SPSS Statistics, version 32.0 (IBM Corp., Armonk, NY, USA). The three studies with non-placebo designs were synthesized narratively (see Section 3).

Each study’s weight for pooling was calculated as the inverse of its variance (1/SE^2^); full details of the effect-size calculation, the choice between random-effects and fixed-effect models, and the sensitivity analyses performed are given below, following the description of the I^2^ statistic.

Forest plots were used to graphically present individual and combined effect sizes. Heterogeneity was assessed visually and, where applicable, with the I^2^ statistic and Cochran’s Q test. Cochran’s Q test evaluates whether observed variations in effect sizes across studies are compatible with chance alone. The Q statistic was calculated by summing the weighted squared deviations of each study’s effect size from the pooled effect size, using inverse variance weights (weight = 1/SE^2^). The I^2^ statistic, derived from the Q value, estimates the proportion of total variability among effect estimates that is due to true heterogeneity rather than sampling error. It was calculated as follows:

$$I^{2}=\frac{Q-\left(k-1\right)}{Q}\times 100\%$$

where k is the number of studies included.

An I^2^ value of 0% was interpreted as no observed heterogeneity. If Cochran’s Q was less than or equal to (k − 1), I^2^ was set to zero following standard practice.

For the five placebo-controlled RCTs reporting a continuous instrumental (EMG-based) measure of muscle activity, effect sizes were computed as Hedges’ g from group means, standard deviations, and sample sizes at each study’s primary post-treatment time point; where a study’s design (crossover or within-subject change-score) did not permit this directly, the standardized mean difference was instead derived from the reported mean difference and its associated test statistic, under explicitly stated approximating assumptions detailed in the Results. A random-effects model (DerSimonian–Laird) [37] was used as the primary pooling method given the anticipated methodological and clinical heterogeneity across studies; a fixed-effect (inverse-variance) model was computed as a sensitivity analysis, together with Cochran’s Q, I^2^, and a leave-one-out sensitivity analysis. The DerSimonian–Laird estimator was selected, rather than alternatives such as restricted maximum likelihood, because it remains the most widely used and readily interpretable random-effects estimator for pooling a small number of studies (k = 5) and was specified a priori; formal statistical tests for small-study effects (e.g., Egger’s regression test, funnel-plot asymmetry) were not performed, consistent with the standard methodological guidance that such tests are unreliable and likely underpowered with fewer than 10 studies per outcome. Because the included instrumental measures did not all quantify the same construct of muscle activity—some capturing the frequency of bruxism events and others the amplitude/intensity of the EMG signal—and because a standardized mean difference formally assumes a shared underlying construct, studies were additionally classified post hoc into these two construct subgroups, each pooled separately with a fixed-effect model, with a formal test for the difference between subgroups. This subgroup comparison is explicitly exploratory, given the small number of studies per subgroup, and is intended to make construct-related heterogeneity transparent rather than to provide a definitive comparison. Outcomes assessed by questionnaires, clinical global impression scales, or other non-instrumental methods were not amenable to this quantitative synthesis and were instead summarized narratively (see Section 3), alongside the three studies with non-placebo designs.

## 3. Results

### 3.1. Study Selection and Characteristics

Of 1128 records identified through the literature search (covering the literature through July 2026), 8 studies met the eligibility criteria and were included in this review (Figure 1 displays the PRISMA flowchart). Verification against the primary source of each included study showed that one of them, Shim et al. (2014) [38], compares two active BoNT-A regimens (masseter-only versus masseter-plus-temporalis) without a placebo or untreated arm; it has therefore been reclassified, together with two other studies with non-placebo designs, as synthesized narratively rather than pooled quantitatively. Five placebo-controlled randomized controlled trials [39,40,41,42,43] were included in the quantitative meta-analysis, encompassing a total of 101 participants (Cruse et al. [39] contributed 22 participants in a crossover design in which each participant received both active and placebo phases; the remaining four studies contributed 43 participants treated with botulinum toxin type A (BoNT-A) and 36 receiving placebo across parallel-group designs). Sample sizes per study ranged from 12 (6 per arm) to 23 participants. Four studies [40,41,42,43] evaluated the effect of BoNT-A injections compared to placebo on masticatory muscle activity, assessed by electromyography (EMG) or a Bruxism Index (BI) derived from EMG, while Cruse et al. [39] additionally analyzed various combinations of infiltrations of the masticatory muscles within its placebo-controlled crossover design. Three studies were synthesized narratively because their designs were not compatible with quantitative pooling: Shim et al. (2014) [38] (n = 20) and an active-comparator RCT [44] (n = 30) each compared two active BoNT-A regimens targeting different muscle combinations without a placebo arm, while a large real-world observational study [45] (n = 304) assessed masseter EMG amplitude before and after BoNT-A injection without any control group. All three studies are summarized narratively alongside the pooled analysis below. Across all 8 included studies, a total of 455 participants contributed EMG data (101 in the pooled placebo-controlled analysis; 354 in the narrative synthesis). Follow-up durations across all 8 studies ranged from 2 weeks to 6 months. Sample sizes reported here reflect the number of participants with analyzed EMG data at the relevant time point, which in three studies [39,41,43] was smaller than the number originally randomized due to post-randomization attrition (detailed in Table 2). Study characteristics are displayed in Table 2.

### 3.2. Diagnostic Criteria for Bruxism Across Included Studies

Bruxism diagnosis varied considerably in rigor across the eight included studies, spanning the full range of the possible/probable/definite grading described in the Introduction [1]. Cruse et al. [39] and Shim et al. [43] applied the most instrumentally rigorous, “definite”-tier criteria: Cruse et al. required a positive surface-EMG-derived Bruxism Index (BI, average bruxism events/hour of sleep) above a pre-specified threshold (BI > 5) for enrolment, while Shim et al. required audio-video-polysomnographic (PSG) confirmation of SB scored according to American Academy of Sleep Medicine criteria. Ondo et al. [41] also required PSG-confirmed SB for enrolment, but used EMG only as an exploratory endpoint, with clinical global impression as the trial’s primary outcome. Shim et al. (2014) [38] and Alzaeem et al. [44] used a clinical diagnosis of SB supported by validated questionnaires and, in the latter, EMG-documented muscle hyperactivity plus erosive tooth wear signs (a “probable”-tier composite). Sherhi et al. [42] diagnosed nocturnal bruxism clinically, based on self-reported grinding/clenching plus at least one supporting sign (occlusal wear, audible grinding, or jaw muscle discomfort). Lee et al. [40] relied on self-report alone for study entry (a “possible”-tier diagnosis), using EMG only as the outcome measure rather than as a diagnostic gate. Furuhata et al. [45], the additional observational study, used the most comprehensive clinical protocol, combining an 11-item checklist (occlusal wear, appliance damage, tenderness, partner-reported grinding, and, when available, PSG) classified as “probable” or “definite” bruxism per the international consensus criteria [1]. This heterogeneity in diagnostic rigor is a relevant methodological consideration: studies enrolling participants on self-report or clinical grounds alone [38,40,42] may include a less homogeneous bruxism population than those requiring instrumental (EMG/PSG) confirmation [39,41,43], which could contribute to between-study variability in baseline muscle activity and, potentially, in the magnitude of the measured EMG response to BoNT-A; this is revisited in Section 4.

### 3.3. Evaluation of Awake vs. Sleep Bruxism

All eight included studies focused primarily on sleep bruxism (SB); none provided a fully separate quantitative analysis of awake bruxism (AB) muscle activity. Cruse et al. [39], Lee et al. [40], Shim et al. [38,43], and Ondo et al. [41] confirmed SB using overnight surface EMG or full polysomnography. Sherhi et al. [42] and Alzaeem et al. [44] diagnosed nocturnal (sleep) bruxism clinically, supplemented by daytime EMG recordings rather than overnight monitoring. Furuhata et al. [45], the observational study, enrolled patients with a mixed clinical picture of sleep and/or awake bruxism (masticatory muscle pain or fatigue “upon waking or during the day”), but reported masseter EMG amplitude as a single pooled measure without AB/SB stratification. Awake bruxism was only indirectly captured, via self-report questionnaire items, in Ondo et al. [41] and Sherhi et al. [42]. Taken together, the current evidence base on the EMG effect of BoNT-A remains almost exclusively derived from SB populations; dedicated AB studies with instrumental (EMG) confirmation are needed.

### 3.4. Electromyographic Activity Reduction

Effect sizes for the EMG-based muscle activity outcome (masseter/temporalis EMG amplitude, bruxism index, or bruxism-related EMG events, depending on what each study reported) were computed as Hedges’ g using group means, standard deviations, and sample sizes at each study’s primary post-treatment timepoint (typically 2–4 weeks): Lee et al. [40] (EMG events/hour), Sherhi et al. [42] (masseter EMG amplitude), and Shim et al. [43] (peak EMG amplitude) permitted this computation directly. For two studies whose design did not permit a direct group-mean comparison, an approximation was required: Cruse et al. [39], a crossover trial, reported only a paired mean difference (−1.66 Bruxism Index events/hour, active vs. placebo phase) and its F-statistic; the standardized mean difference was derived by dividing this mean difference by the outcome’s baseline standard deviation. Ondo et al. [41] reported EMG-derived bruxing events only as pre-post changes in each arm rather than as a between-group comparison at a single time point; the standardized mean difference was derived from the between-group difference in these change scores, using their pooled standard deviation under a conservative assumption of zero correlation between each participant’s baseline and follow-up measurement (which likely underestimates the true precision of a paired analysis). Both derivations are approximations necessitated by the summary statistics available in the published reports, rather than exact recalculations from raw data, and are flagged accordingly in the Limitations.

For Ondo et al. [41], the change-score pooled standard deviation was recalculated at each assumed correlation from the reported pre- and post-treatment means and SDs for each arm (BoNT-A: 9.18 ± 8.48 to 6.95 ± 7.04/h, n = 13; placebo: 4.63 ± 3.45 to 10.65 ± 9.57/h, n = 9), yielding g = −0.74 (95% CI: −1.59 to 0.11) at r = 0 (the conservative assumption used in the main analysis), rising in magnitude to g = −0.86 at r = 0.3, −0.98 at r = 0.5, −1.17 at r = 0.7, and −1.56 at r = 0.9 (see Supplementary Material S1). For Cruse et al., we additionally computed a paired-difference standardized effect (mean difference divided by the SD of the within-subject active–placebo difference, itself a function of r, using the reported 4-week active and placebo SDs of 4.09 and 4.11 and n = 22), which is arguably more appropriate for a crossover design than the baseline-SD standardizer used in the main analysis: this yielded g = −0.29 at r = 0, −0.34 at r = 0.3, −0.40 at r = 0.5, −0.52 at r = 0.7, and −0.91 at r = 0.9, bracketing the baseline-SD estimate reported above (g = −0.58, which corresponds to an implied correlation of approximately 0.6–0.65 under this alternative parameterization) (see Supplementary Material S2). Re-pooling the frequency/events subgroup (Cruse, Lee, Ondo) by fixed-effect inverse-variance weighting using these alternative Cruse and Ondo estimates, holding Lee et al. [40] fixed, shifted the subgroup pooled estimate from g = −0.50 (95% CI: −0.87 to −0.14) at r = 0 to g = −1.16 (95% CI: −1.58 to −0.74) at r = 0.9, compared with g = −0.70 (95% CI: −1.04 to −0.37) originally reported for this subgroup using the zero-correlation/baseline-SD approximations (see Supplementary Material S3). Across the entire plausible range of correlations tested (0 to 0.9), the pooled frequency-subgroup estimate remained negative, and its 95% CI excluded zero at every tested correlation, including r = 0, even though the individual-study CIs for Ondo (and, at low r, Cruse) crossed zero in isolation. The magnitude of the pooled effect is therefore sensitive to the unreported correlation—ranging roughly two-fold across plausible assumptions—but its direction and statistical significance at the subgroup level are not, supporting the qualitative conclusion that BoNT-A reduces bruxism-event frequency while cautioning against over-interpreting the precise magnitude of g = −0.70 reported as the primary frequency-subgroup estimate.

**Figure 2:**
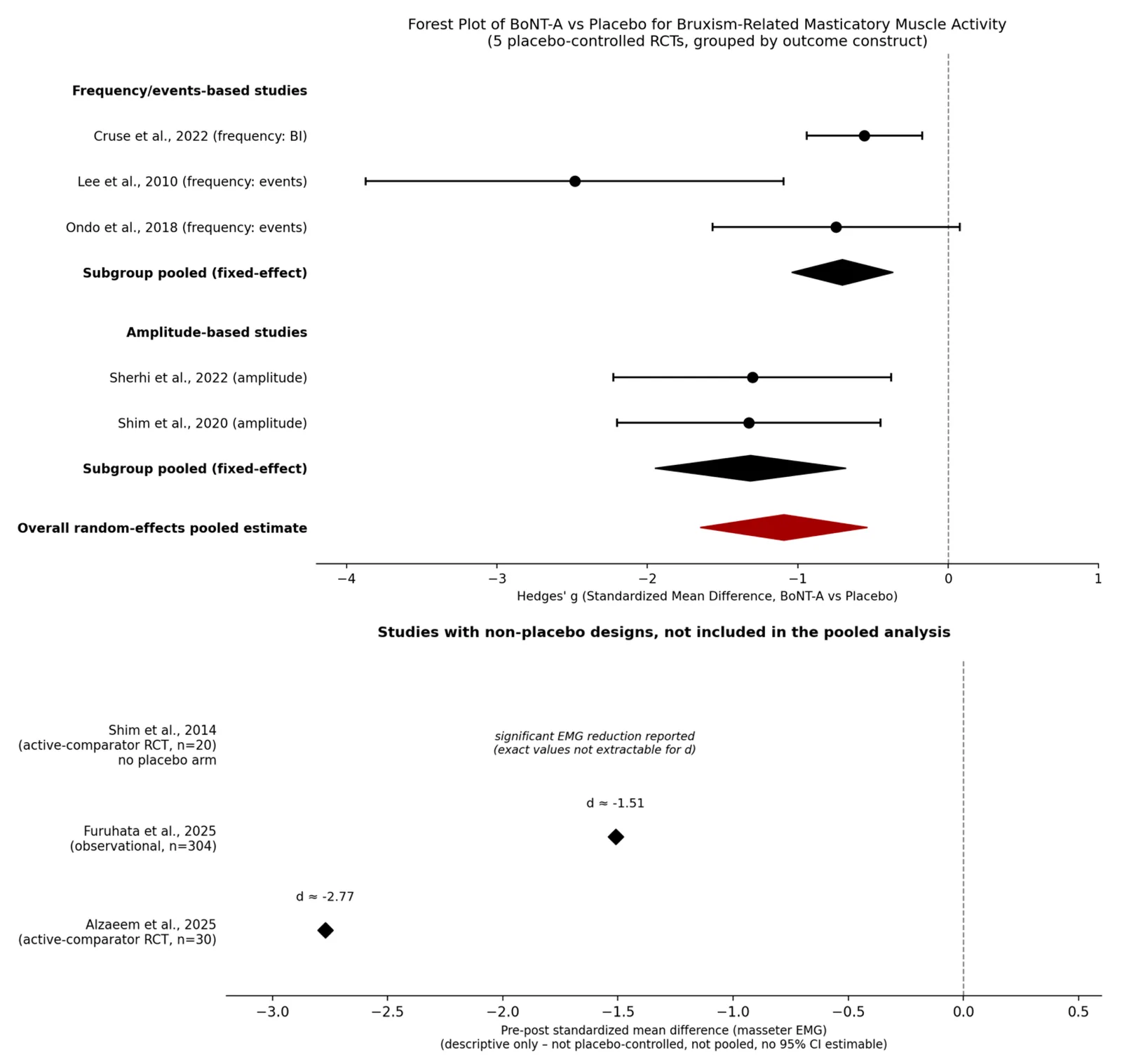
Forest plot showing the individual effect sizes (Hedges’ g with 95% CI) for the 5 placebo-controlled RCTs [38,39,40,41,42,43]. analyzing EMG-based muscle activity reduction following BoNT-A treatment, recalculated directly from each study’s reported means, standard deviations, and sample sizes, and grouped by outcome construct (top panel). Black diamonds show the fixed-effect subgroup pooled estimates for the frequency/events-based studies (g = −0.70) and the amplitude-based studies (g = −1.31); the dark red diamond shows the overall random-effects pooled estimate across both constructs (g = −1.09, 95% CI: −1.65 to −0.54), which should be interpreted in light of the construct heterogeneity discussed in Section 2 and Section 3. Shim et al. (2014) [38] is shown separately (see the bottom panel) because, on verification against its primary source, it was found to compare two active BoNT-A regimens without a placebo arm and was therefore excluded from pooling. The bottom panel displays, for descriptive comparison only, pre-post standardized mean differences (where computable from published summary statistics) for the three studies with non-placebo designs—Shim et al. (2014) [38], Alzaeem et al. (2025) [44], and Furuhata et al. (2025) [45]—none of which were included in the pooled estimate.

All five placebo-controlled studies reported a reduction in masticatory muscle EMG activity following BoNT-A injections relative to placebo, with individual Hedges’ g ranging from −0.56 to −2.48 (Figure 2, top panel; Table 2 reports each study’s primary EMG-based measure). Although Ondo et al. [41] reported a numerically greater reduction in EMG-recorded bruxing events in the BoNT-A group, this did not reach statistical significance, consistent with EMG having been an exploratory rather than primary endpoint in that trial. Cochran’s Q was 9.65 (df = 4, *p* = 0.047), and I^2^ was 58.6%, indicating moderate-to-substantial heterogeneity; a random-effects (DerSimonian–Laird) model was therefore used as the primary analysis. The random-effects pooled estimate was g = −1.09 (95% CI: −1.65 to −0.54), indicating a statistically significant pooled reduction in EMG-measured muscle activity with BoNT-A compared with placebo (interpreted with the construct-heterogeneity caveat below). A fixed-effect model, computed as a sensitivity analysis, yielded a smaller but still large and significant pooled estimate (g = −0.84, 95% CI: −1.13 to −0.54), reflecting the down-weighting of the fixed-effect model on Lee et al. [40], the smallest and most extreme study. Leave-one-out sensitivity analysis confirmed the robustness of the random-effects pooled estimate: removing any single study shifted the pooled g only modestly, within a range of −0.92 (excluding Lee et al. [40]) to −1.34 (excluding Cruse et al. [39]), and the effect remained large and statistically significant in every iteration. All five studies concerned sleep bruxism (SB); due to limited reporting, a separate pooled analysis for awake bruxism (AB) could not be conducted.

Because the overall pooled estimate combines two conceptually different EMG constructs (event frequency and signal amplitude; see Section 2), an exploratory subgroup analysis compared them directly. The three frequency/events-based studies (Cruse et al. [39], Lee et al. [40], Ondo et al. [41]) yielded a fixed-effect pooled g of −0.70 (95% CI: −1.04 to −0.37), whereas the two amplitude-based studies (Sherhi et al. [42], Shim et al. [43]) yielded a fixed-effect pooled g of −1.31 (95% CI: −1.95 to −0.68)—a numerically larger effect. The test for subgroup difference was not statistically significant (Q-between = 2.77, df = 1, *p* = 0.096), but with only two and three studies per subgroup, this comparison is markedly underpowered, and the numerical difference should be regarded as hypothesis-generating rather than conclusive. Practically, this means the overall pooled g = −1.09 reported above is best understood as a summary across two related but distinct signals—BoNT-A reduces both how often bruxism events are detected on EMG/PSG and how intensely the muscle contracts during those events—rather than as a single, unambiguous magnitude of “muscle activity reduction.”

The forest plot (Figure 2, top panel) presents the individual effect sizes and 95% CIs for the five placebo-controlled studies, grouped visually by outcome construct, together with the overall random-effects pooled diamond. The bottom panel separately displays Shim et al. (2014) [38] alongside the two other non-pooled studies (see below and Section 2).

Beyond the five pooled RCTs, the two additional non-placebo-controlled studies extend this evidence to a further 334 patients (Table 2) with independent, non-pooled confirmation of the same EMG-reducing effect. The active-comparator RCT [44] shows this most directly: bilateral masseter BoNT-A injection (15 U/side) reduced masseter EMG amplitude from baseline to 2 weeks in both study arms (masseter-only and masseter-plus-lateral-pterygoid, 10 U/side), with no significant between-group difference in masseter EMG reduction (*p* > 0.05); the group additionally injected in the lateral pterygoid muscle showed a significantly greater reduction in lateral pterygoid EMG amplitude (*p* < 0.001). The large observational study [45] (n = 304, bilateral masseter injection, 25 U/side) reported a reduction in mean masseter EMG amplitude from 189.0 (SD 120.5) µV at baseline to 55.4 (SD 32.9) µV at 2 months (*p* < 0.001, Wilcoxon signed-rank test), and identified age, sex, and baseline EMG amplitude as significant predictors of the magnitude of EMG reduction. Neither study included a placebo or untreated comparison group, so their effect estimates are not directly comparable to the placebo-controlled SMD reported above; they are reported here as independent, real-world and alternative-protocol evidence corroborating the direction and approximate magnitude of the EMG-reducing effect of BoNT-A on the masseter muscle. For descriptive visualization only (Figure 2, bottom panel), we additionally calculated a pre-post standardized mean difference for each of these two studies from their reported baseline and follow-up masseter EMG means and SDs, using the pooled pre/post SD as the standardizer (d ≈ −1.51 for Furuhata et al. [45]; d ≈ −2.77 for Alzaeem et al. [44], combining both study arms). Because the correlation between each participant’s baseline and follow-up measurement was not reported, a 95% CI could not be estimated for these values, and their markedly larger magnitude relative to the placebo-controlled random-effects pooled estimate (g = −1.09) most likely reflects the absence of a placebo/control arm (e.g., regression to the mean, natural fluctuation) rather than a truly larger treatment effect—which is precisely why they were kept out of the quantitative pooling. Taken as a whole, all eight included studies—spanning five placebo-controlled RCTs, two additional active-comparator RCTs, and one large observational series, together contributing 455 participants—point in the same direction: BoNT-A injection into the masseter (±other masticatory muscles) consistently and substantially reduces EMG-recorded muscle activity, regardless of study design, diagnostic rigor, dosing protocol, or population size.

### 3.5. Change in Questionnaire-Based, Clinical Global Impression, and PSG-Frequency Measures of Bruxism Activity

Beyond EMG-based measures, six of the eight included studies also assessed bruxism activity using questionnaires, clinical global impression (CGI) scales, or polysomnography (PSG)-scored episode-frequency parameters, and these results were considerably less consistent than the instrumental EMG findings above. Ondo et al. [41] found that CGI ratings of “much/very much improved” and a bruxism VAS both significantly favored BoNT-A over placebo (both *p* < 0.05)—the clearest subjective/clinician-rated signal in the entire evidence base, and notably stronger, statistically, than this same trial’s exploratory EMG finding. In contrast, Cruse et al. [39] found no significant difference between active and placebo phases on a bruxism symptom questionnaire at 4 or 12 weeks, despite the significant objective BI reduction described above; the study authors themselves highlighted this discrepancy between instrumentally measured bruxism severity and questionnaire-based bruxism frequency. Lee et al. [40] similarly found that bruxism symptom scores improved significantly over time in both the BoNT-A and saline groups, with no significant time-by-group interaction (*p* = 0.362), indicating no BoNT-A-specific subjective benefit despite the BoNT-A-specific reduction in EMG-derived bruxism events in the same trial. Among the non-placebo-controlled studies, Shim et al. (2014) [38] found self-reported reduction in tooth grinding in only 9 of 20 participants, with no significant difference between the masseter-only and masseter-plus-temporalis groups, and Alzaeem et al. [44] found that a four-item bruxism questionnaire improved substantially in both muscle-targeting protocols at 2 weeks before gradually returning toward baseline by 6 months, again with no significant between-group difference at any time point. Shim et al. (2020) [43], using PSG scoring, found no significant reduction in the frequency, number of bursts, or duration of rhythmic masticatory muscle activity (RMMA) episodes with BoNT-A relative to placebo, even though the amplitude of those same episodes was significantly reduced (see above)—indicating that, at least in this trial, BoNT-A altered the intensity but not the generation of bruxism episodes. Taken together, these questionnaire-, CGI-, and PSG-frequency-based findings do not show a consistent BoNT-A-specific effect across studies, in contrast to the more consistent instrumental EMG evidence (which, as noted above, nonetheless combines two related but distinct constructs); only Ondo et al. [41] reported a clear subjective advantage for BoNT-A, while Cruse et al. [39], Lee et al. [40], Alzaeem et al. [44], and Shim et al. (2020) [43] each found that a measure of bruxism frequency or self-reported symptoms did not differentiate BoNT-A from its comparator, even in trials where an EMG-based measure from the same participants did. Furuhata et al. [45] did not employ a comparable bruxism-frequency questionnaire and is therefore not included in this comparison; its reported improvements in sleep quality, shoulder/neck stiffness, and headache are symptom/quality-of-life measures outside the scope of this review (see Section 2).

### 3.6. Protocol and Administration of Botulinum Toxin Injection

Doses ranged from 20U to 200U in total per subject across the five placebo-controlled RCTs. All studies used bilateral injection into the masseter; two studies [39,41] also included the temporalis, and one study [39] also injected the medial pterygoid muscle. Most studies used standard dilution (1–2 mL of sterile saline solution/100U). Injection depth was typically intramuscular; only Cruse et al. [39] used EMG guidance for the masseter and medial pterygoid, and all the others [40,41,42,43] performed injection using anatomical landmarks. It is not feasible to ascertain a common number of infiltrations for each masticatory muscle: the masseter has been infiltrated by Cruse et al. [39] and Ondo et al. [41] using multiple sites, as illustrated in Table 2. Most of the placebo-controlled studies assess the effects of masseter infiltration in isolation [40,42,43], whereas Cruse et al. [39] and Ondo et al. [41] compare masseter (± temporalis ± medial pterygoid) infiltration against placebo. Cruse et al. [39] additionally conducted a within-trial comparison of different combinations of masticatory muscle infiltration (masseter versus masseter plus temporalis versus masseter plus temporalis plus medial pterygoid), finding no significant between-group difference in efficacy on BI, though a numerically greater effect was seen with more muscles/higher total dose. Three further studies with non-placebo designs directly compared different muscle-targeting strategies for BoNT-A: Shim et al. (2014) [38] compared masseter-only versus masseter-plus-temporalis injection (no significant between-group difference in EMG reduction), and the active-comparator study [44] similarly found no significant between-group difference in masseter EMG reduction when the lateral pterygoid muscle was additionally injected, though that muscle’s own EMG activity was, expectedly, more reduced when directly targeted. Across all studies that compared different muscle combinations (placebo-controlled or not), the consistent finding is that adding further muscles to the masseter injection did not meaningfully change the masseter’s own EMG response, but did reduce activity specifically in the co-injected muscle. Furuhata et al. [45], the large observational study, did not compare muscle combinations; it administered a single, standardized masseter-only protocol (≈25U per side, three sites, anatomical landmarks, no EMG or ultrasound guidance) to all 304 patients, and its results indicate that this masseter-only, non-guided approach—the most conservative and widely generalizable of all eight protocols—was sufficient to produce a substantial real-world reduction in masseter EMG amplitude, consistent with the placebo-controlled and active-comparator evidence that additional muscles are not required to reduce masseter activity itself.

### 3.7. Side Effects

Mild adverse events were reported in three of the five placebo-controlled RCTs, including temporary facial asymmetry [39], discomfort at the injection site [42], and cosmetic deficits in the smile [41]. No serious adverse effects were reported within the follow-up period of any of these studies. All side effects are detailed in Table 3. The three studies with non-placebo designs reported no serious adverse events either: Shim et al. (2014) [38] and the active-comparator RCT [44] did not report muscle-related complications beyond expected transient effects, and the observational study [45] reported no obvious complications among 304 patients, consistent with a favorable short-term safety profile across the broader evidence base.

### 3.8. Risk of Bias Assessment

Most of the five placebo-controlled RCTs were low risk across domains; Cruse et al. [39] had some concerns specifically regarding the randomization domain (allocation concealment for the crossover order was not fully detailed), despite otherwise rigorous double-blinding of participants, injecting clinician, and outcome assessors. See Table 4 for details. Among the three non-placebo studies, Shim et al. (2014) [38] had some concerns regarding randomization to muscle group and lack of blinding; the active-comparator RCT [44] was assessed as high risk for randomization, given allocation by an unconcealed coin toss, and as low risk for outcome assessment (blinded EMG evaluators), but, as an open-label surgical procedure, could not blind the treating clinician. The observational study [45] was evaluated with the Newcastle–Ottawa Scale and rated as satisfactory for outcome ascertainment (standardized EMG equipment, pre-defined measurement protocol) but was limited by the absence of a control group, a limitation inherent to its design.

### 3.9. Outcome-Specific Risk-of-Bias Domain Judgments (RoB 2)

Cochrane RoB 2 signaling questions were applied to the methodological details reported in each primary publication, as follows for the EMG-based outcome analyzed in this review from each RCT:

Cruse et al. [39] (BI events/hour; crossover): D1—Some concerns (randomization to injection-order and dose group described only as “randomized”, with no detail on sequence generation, though drug/placebo preparation was concealed by blinded pharmacists); D2—Low (full double-blind design; injecting clinician blinded to content); D3—Some concerns (substantial attrition: 22/35 randomized participants analyzed, ~37% loss, reasons given but balance across arms not detailed); D4—Low (BI derived from EMG analyzed by a single scientist blinded to treatment arm and symptoms); D5—Low (prospectively registered, ACTRN12618001430224; reported outcomes match registration). Overall: Some concerns.

Lee et al. [40] (masseter EMG events/hour): D1—Some concerns (randomization method not described); D2—Low (subjects, operators, and data collectors all blinded to injected material); D3—Low (no withdrawals, complete data); D4—Low (EMG analyzed off-line by a pre-specified semi-automated method); D5—Some concerns (no evidence of prospective registration, consistent with standard practice at the time of publication, 2010). Overall: Some concerns.

Ondo et al. [41] (PSG bruxing events/hour): D1—Low (explicit random-number-generator allocation by a coordinator not otherwise involved in the trial); D2—Some concerns (blinding of the injecting clinician to treatment content not explicitly described); D3—Some concerns (one placebo-arm dropout after randomization; one participant per group missing a final polysomnogram, i.e., missing data specifically affecting the EMG/PSG outcome analyzed here); D4—Some concerns (blinding of the specific reviewer of PSG bruxism-event data to treatment allocation not explicitly stated, unlike the clearly blinded assessment of the CGI/VAS outcomes); D5—Low (prospectively registered, NCT00908050; reported outcomes match registration). Overall: Some concerns.

Sherhi et al. [42] (masseter EMG amplitude): D1—Low (computer-generated randomization with numbered, opaque, sealed envelopes); D2—Some concerns (principal investigator/injecting clinician not blinded to allocation, by the authors’ own statement); D3—Low (minimal, balanced attrition: 1 dropout per group of 11); D4—Some concerns (blinding of the EMG outcome assessor not explicitly described); D5—Low (prospectively registered, NCT05620316). Overall: Some concerns.

Shim et al. [43] (peak EMG amplitude during RMMA)**:** D1—Some concerns (randomization method not described); D2—Low (patients blinded; saline placebo comparator); D3—Some concerns (7/30 participants, ~23%, unavailable for follow-up PSG; balance across arms not detailed); D4—Low (two independent, blinded observers scored recordings in randomized order); D5—Low (prospectively registered, KCT0004459). Overall: Some concerns.

For the two non-placebo-controlled RCTs synthesized narratively: Shim et al. (2014) [38]—D1: Some concerns (randomization method not described); D2: Low (blinded video-PSG scoring; both arms received active BoNT-A, minimizing performance-bias concerns); D3: Low (2 dropouts and 2 further baseline exclusions from an initial 24, adequately explained); D4: Low (blinded observer scoring); D5: Some concerns (no evidence of prospective registration). Overall: Some concerns. Alzaeem et al. [36]—D1: High (allocation by coin toss, an unconcealed and potentially predictable method with no allocation-concealment mechanism described); D2: Some concerns (the treating surgeon was explicitly not blinded, by the authors’ own statement, though outcome assessors were blinded, ICC > 0.8); D3: Low (no dropouts; 30/30 analyzed); D4: Low (EMG outcome assessed by blinded evaluators); D5: Some concerns (the trial was registered, ISRCTN13377883, but the registration date of 20 December 2024 postdates the stated study period of January–November 2024, raising a concern about retrospective registration). Overall: High, driven by the randomization method.

These outcome-specific judgments are broadly consistent with, and provide the detailed justification underlying, the study-level overall judgments already summarized in Table 4. Both the RoB 2 domain judgments above (Section 3.9) and the GRADE domain-specific rationale presented below (Section 3.10) were independently evaluated by two reviewers (M.V. and A.C.), who discussed their assessments and reached the same conclusions.

### 3.10. GRADE Certainty of Evidence—Domain-Specific Rationale

Building on the risk-of-bias judgments above and the heterogeneity statistics already reported (I^2^ = 58.6% for the combined EMG-based estimate), the explicit domain-specific GRADE reasoning for these three outcomes are: (1) EMG-based muscle activity magnitude (frequency and amplitude constructs combined): starting point is high (randomized evidence); downgraded one level for risk of bias (the majority of contributing RCTs were rated “some concerns” on RoB 2, predominantly for randomization-method reporting and missing outcome data); downgraded one further level for inconsistency (I^2^ = 58.6%, moderate-to-substantial heterogeneity, compounded by the frequency-versus-amplitude construct heterogeneity described above); not downgraded for indirectness (populations, interventions, and comparators are directly applicable); not further downgraded for imprecision at this combined level, given the reasonably narrow overall confidence interval. Resulting certainty: low. (2) Questionnaire-, CGI-, and PSG-frequency-based bruxism outcomes: starting point is high; downgraded for risk of bias (some concerns across contributing studies); downgraded for inconsistency (only 1 of 6 studies assessing these outcomes found a clear BoNT-A-specific benefit, with the remainder showing no significant difference from comparator); downgraded for imprecision (each outcome type analyzed narratively in only one or two studies, with small samples and wide, uncertain estimates, precluding pooling). Resulting certainty: very low. (3) Short-term safety: starting point is high; downgraded for imprecision (small number of controlled participants and low absolute event counts across the pooled placebo-controlled trials); downgraded for indirectness (heterogeneous dosing protocols and injected muscles across studies limit the applicability of a single pooled safety statement); not further downgraded for risk of bias, as adverse-event ascertainment was reasonably systematic (structured recording at each study visit) across the contributing trials. Resulting certainty: low. Publication bias was not formally assessed for any outcome (e.g., via funnel plot or Egger’s test) given the small number of contributing studies (k = 5 for the quantitative synthesis), consistent with standard methodological guidance that such tests are unreliable with fewer than 10 studies.

The formal GRADE Summary of Findings, consolidating the domain-specific rationale above, is provided in Table 5.

## 4. Discussion

This systematic review provides converging, but methodologically heterogeneous, evidence that BoNT-A injections into the masticatory muscles reduce EMG-detectable muscle activity in SB. Because the pooled placebo-controlled evidence spans two related but distinct constructs, the construct-specific estimates—g = −0.70 for event frequency and g = −1.31 for EMG signal amplitude (see Section 3)—are regarded as the primary quantitative findings of this review, while the composite estimate below is reported as a secondary, exploratory summary. Pooling five placebo-controlled RCTs [39,40,41,42,43] under a random-effects model, which was preferred given moderate-to-substantial heterogeneity (I^2^ = 58.6%), yielded a statistically significant secondary, composite pooled effect in favor of BoNT-A over placebo (Hedges’ g = −1.09, 95% CI: −1.65 to −0.54). As already noted, the formal test for this construct-specific difference was itself underpowered and non-significant (see Section 3). The overall pooled estimate should therefore be read as a summary signal across related but non-identical outcome constructs, not as a single precise effect size for a unitary construct of “muscle activity.” These findings nonetheless align with previous reports that BoNT-A administration reduces measurable masticatory muscle overactivity [22,23], and are corroborated in direction, though not in exact magnitude, by three additional non-placebo-controlled sources: Shim et al. (2014) [38], a large real-world observational series [45], and an active-comparator RCT [44], all three of which documented a reduction in masseter/temporalis EMG amplitude after BoNT-A injection, without a placebo comparator.

Several mechanisms underpin BoNT-A’s efficacy. By inhibiting acetylcholine release at the neuromuscular junction, BoNT-A reduces involuntary muscle contractions, which is reflected in decreased EMG amplitude post-injection, as seen in studies like Shim et al. (2020) [43] and Lee et al. [40]; a pattern of EMG-amplitude reduction after BoNT-A is also reported in broader masticatory-muscle-pain populations [46,47], and, at a larger scale and outside the placebo-controlled synthesis, in the observational series by Furuhata et al. [45]. This EMG-based reduction is corroborated by direct morphometric evidence: a recent prospective study using 3D structured-light facial scanning documented a significant bilateral reduction in masseter surface volume as early as one month after BoNT-A injection in patients with myofascial pain and bruxism, with the effect sustained at three months [48]. Because this review’s outcome is restricted to bruxism/muscle activity, these findings should be interpreted strictly as evidence about whether BoNT-A achieves its proposed peripheral mechanistic target—a reduction in masticatory muscle hyperactivity—and not as evidence, one way or another, about its effect on pain, function, or other bruxism-related symptoms, which fall outside the scope of the outcomes evaluated here.

An important corollary of this mechanistic interpretation concerns the level at which BoNT-A acts. Bruxism, whether expressed during sleep or wakefulness, is now understood to be generated primarily by central mechanisms—including brainstem motor pattern generators, transient sleep arousals, and neurotransmitter systems—rather than by a primary abnormality of the masticatory muscles themselves [4,5]. BoNT-A, in contrast, acts exclusively at the peripheral neuromuscular junction, chemically denervating the injected muscle fibers without modifying the central motor program that drives bruxism [24,49]. Consistent with this, De la Torre Canales et al. and, more recently, the umbrella review by Coelho et al. concluded that BoNT-A reduces the intensity of masticatory muscle contractions during bruxism episodes rather than their generation or frequency [24,49,50]—a distinction that closely mirrors both the amplitude-versus-frequency dissociation identified in the construct-based subgroup analysis of the present review (see above) and the lack of a consistent BoNT-A effect on PSG-scored RMMA episode counts in Shim et al. (2020) [43]. Because the central motor engram driving bruxism is left unmodified, the underlying tendency to brux would be expected to persist unchanged once BoNT-A’s peripheral effect wears off, typically within three to six months [18,51]; some animal data suggest a possible, still poorly characterized central contribution to BoNT-A’s action after intramuscular injection, but this remains an unconfirmed hypothesis rather than an established clinical mechanism [51]. Clinically, this reinforces that BoNT-A should be understood as a symptomatic, muscle-directed intervention rather than a disease-modifying treatment for bruxism, a distinction previously highlighted in the orofacial pain and movement-disorder literature more broadly [18,52,53].

Even within that restricted scope, however, the evidence is far from uniform once all assessment methods used across the eight studies are considered together, and this divergence is itself an important finding of this review, not a peripheral detail. Only Ondo et al. [41] found a clear BoNT-A-specific benefit on a questionnaire/clinician-rated measure (clinical global impression and a bruxism VAS, both *p* < 0.05). In every other study that assessed bruxism frequency subjectively or by PSG-scored episode counts—Cruse et al. [39] (bruxism symptom questionnaire), Lee et al. [40] (bruxism symptom score), Alzaeem et al. [44] (four-item bruxism questionnaire), and Shim et al. (2020) [43] (PSG-scored RMMA episode frequency, bursts, and duration)—that measure improved similarly in both arms or did not differ significantly between BoNT-A and its comparator, even when an EMG-based measure from the very same participants did show a BoNT-A-specific reduction. This pattern suggests that BoNT-A’s peripheral, muscle-restricted mechanism of action is more reliably captured by direct instrumental measurement of muscle electrical activity than by patient- or clinician-reported measures of bruxism frequency, or by PSG-scored counts of when episodes occur, both of which are plausibly influenced by additional central, behavioral, and expectation-related factors that a peripheral neuromuscular blocker would not be expected to modify. Clinically, this means that a reduction in EMG-measured muscle activity after BoNT-A cannot be assumed to translate into a subjectively or clinically noticeable reduction in bruxism frequency, and the two should not be used interchangeably as markers of treatment response.

The heterogeneity in BoNT-A injection protocols complicates cross-study comparisons. Cruse et al. (2022) [39] administered 120U across the masseter, temporalis, and pterygoid muscles, while others used lower doses or limited injection to the masseter alone; Alzaeem et al. [44] used 15U per masseter side and 10U per lateral pterygoid side. The use of guidance tools, such as EMG or ultrasound, varied, potentially influencing the accuracy and efficacy of muscle targeting. Notably, both the pooled RCT analysis and the additional active-comparator RCT [44] converge on the same finding regarding target muscle selection: adding the lateral pterygoid or temporalis muscle to the masseter injection did not confer a significant additional reduction in masseter EMG activity itself, although it did reduce EMG activity specifically within the co-injected muscle.

The heterogeneity in bruxism diagnostic rigor across studies (from self-report alone in Lee et al. [40], through composite clinical criteria in Sherhi et al. [42], Alzaeem et al. [44], and Furuhata et al. [45], to instrumentally confirmed, PSG- or threshold-based diagnoses in Cruse et al. [39], Ondo et al. [41], and Shim et al. [43]) is a plausible contributor to the between-study variability in baseline EMG values and treatment response observed in this review, alongside the differences in injection protocol discussed above. Studies requiring instrumental (EMG/PSG) confirmation for enrolment likely selected a more homogeneous, higher-baseline-activity bruxism population, which may partly explain their comparatively larger and more consistent EMG reductions after BoNT-A, whereas studies relying on self-report or clinical signs alone may have enrolled a broader, more heterogeneous population with lower and more variable baseline muscle activity. This distinction is independent of, and additional to, the placebo-controlled versus non-placebo-controlled distinction already discussed above, and future trials would benefit from adopting a uniform, instrumentally verified diagnostic threshold to reduce this source of heterogeneity.

Taken together, the picture that emerges when all assessment methods used across the eight included studies are considered is one of convergence at the instrumental level and divergence at the questionnaire/clinical level. Every study that measured bruxism/muscle activity by EMG reported a reduction after BoNT-A, and this direction of effect was consistent across placebo-controlled RCTs, two active-comparator RCTs, and a large observational series, regardless of design, diagnostic rigor, or dosing protocol—a genuine strength of the evidence base. However, the precise magnitude of this instrumental effect is considerably less certain than the pooled g = −1.09 figure might suggest in isolation, both because it blends frequency- and amplitude-based EMG constructs (above), and because, as shown in the preceding paragraph, measures of bruxism frequency obtained by questionnaire, clinical impression, or PSG scoring did not consistently move in step with the EMG findings from the same studies. This evidence base therefore does not, by itself, establish whether or how a reduction in EMG-detectable muscle activity translates into a bruxism-frequency reduction that patients or clinicians can perceive, let alone into broader clinical benefit (e.g., reduced pain or improved function), which requires dedicated outcome measures and falls outside the scope of the present review.

### 4.1. Clinical Implications

Based on the current evidence, BoNT-A injections into the masticatory muscles consistently and substantially reduce EMG-detectable masticatory muscle activity, which may make BoNT-A a reasonable adjunctive option to consider for patients with moderate to severe sleep bruxism who have not responded adequately to conservative measures such as occlusal splints or behavioral therapy; because pain, functional limitation, and quality of life were outside the scope of this review, this statement should not be read as evidence of clinical or symptomatic benefit, which requires dedicated investigation. The consistent direction of EMG-activity reduction across placebo-controlled RCTs, two active-comparator RCTs, and a large observational series suggests a mechanistically targeted treatment option for muscle hyperactivity, although the precise magnitude of this effect varies depending on which EMG-based construct (event frequency vs. signal amplitude) is used to quantify it. Clinicians should be aware that this EMG-measured benefit does not reliably track patient- or clinician-reported measures of bruxism frequency, which improved similarly regardless of treatment allocation in most studies that assessed them (see Section 4); EMG or PSG monitoring, rather than symptom report alone, is therefore recommended for objectively verifying treatment response, in addition to its role in diagnosis and treatment planning.

However, BoNT-A should be administered with caution, particularly regarding dosage and injection technique. Clinicians should be aware of the temporary nature of its effects and the need for repeated injections [54], which may have long-term implications such as muscle weakening or atrophy [55,56]. Multidisciplinary approaches that integrate BoNT-A with behavioral, dental, and sleep medicine expertise, or with other targeted interventions such as arthrocentesis in appropriate cases [21], are likely to offer the most comprehensive patient care. Further research into cost-effectiveness, quality of life impact, and personalized treatment algorithms is warranted to better define BoNT-A’s role in clinical practice.

### 4.2. Limitations

Despite the overall favorable findings, several limitations must be acknowledged. First, most studies included in this review had relatively small sample sizes and limited statistical power, which may affect the generalizability of the results. Second, the follow-up durations were relatively short (2 weeks to 6 months), limiting our understanding of the long-term efficacy and safety of BoNT-A treatment. Third, the heterogeneity in BoNT-A protocols—such as dosing, number and depth of injection sites, use of guidance techniques, and choice of target muscles—makes it difficult to establish standardized recommendations. Fourth, there was a predominant focus on sleep bruxism; data on awake bruxism specific to muscle activity were limited. Relatedly, questionnaire-, CGI-, and PSG-frequency-based measures of bruxism activity did not consistently corroborate the EMG-based findings within the same studies (see Section 4); readers should not assume that an EMG-measured reduction in muscle activity implies a proportional, or even detectable, reduction in patient- or clinician-perceived bruxism frequency. Fifth, and most importantly, even within the restricted EMG outcome, quantification methods varied across studies (amplitude vs. event frequency); as detailed in the Results and Discussion, this is precisely why we have already presented the construct-specific estimates (g = −0.70 and g = −1.31) as the primary quantitative findings above, with the all-construct pooled estimate (g = −1.09) as a secondary, descriptive summary; future, larger meta-analyses of this literature should aim to pool enough studies per construct to make this comparison statistically well-powered, which the present review, with only two and three studies per subgroup, cannot achieve. Finally, the two additional non-placebo-controlled studies could not be incorporated into the quantitative pooled analysis because neither included a placebo arm; part of the literature search relied on targeted database searching and manual/citation tracking rather than a fully systematic multi-database strategy for the entire period, so the search should not be considered exhaustive, and a complete, formally re-run search across PubMed, Scopus, and Web of Science is recommended before any further update of this review. Verification of each included study against its primary source revealed that Shim et al. (2014) [38] does not include a placebo arm (both study arms received active BoNT-A, differing only in target muscles); it has therefore been reclassified as a non-placebo-controlled study synthesized narratively, and the pooled effect size has been recalculated from the remaining five placebo-controlled RCTs using Hedges’ g extracted directly from each study’s reported means, standard deviations, and sample sizes (or, where only a crossover mean difference or change scores were reported, from the associated test statistic under explicitly stated approximating assumptions; see Section 2). This recalculation should still be independently verified by the study team using the original raw data where available, particularly for Cruse et al. [39] (crossover design) and Ondo et al. [41] (change-score approximation), for which the summary statistics available in the published reports required an approximating assumption to derive a standardized effect size; a correlation-based sensitivity analysis for both studies has been performed and is reported in Section 3.4, and shows the pooled frequency-subgroup estimate remains directionally consistent and statistically significant across the plausible range of assumed correlations (0–0.9), although its magnitude varies accordingly.

### 4.3. Future Directions

Future trials should focus on:

- Standardizing injection protocols (dose, muscles, technique);
- Conducting long-term follow-up studies to assess durability and safety;
- Expanding research on awake bruxism with objective measures;
- Concurrently reporting instrumental (EMG/PSG) and patient- or clinician-reported measures of bruxism frequency within the same trial, using harmonized, validated instruments, so that the relationship (or dissociation) between the two can be formally examined rather than inferred across separate studies;
- Designing future active-comparator and observational studies with a shared placebo or untreated reference arm, so that their EMG effect sizes can be formally pooled with future RCTs rather than only synthesized narratively;
- Evaluating cost-effectiveness and patient-centered outcomes such as quality of life improvements.

BoNT-A’s role should be considered complementary rather than substitutive, integrated into comprehensive, individualized care plans.

## 5. Conclusions

This systematic review demonstrates that BoNT-A injections into the masticatory muscles consistently reduce EMG-detectable muscle activity in patients with sleep bruxism (SB), a finding consistent in direction across all eight included studies regardless of design, diagnostic criteria, or dosing protocol. A random-effects pooling of five placebo-controlled RCTs yielded a statistically significant effect (Hedges’ g = −1.09, 95% CI: −1.65 to −0.54); however, because this estimate combines studies that measured bruxism event frequency and studies that measured EMG signal amplitude—two related but distinct constructs, with a numerically larger effect observed for amplitude-based measures in exploratory subgroup analysis—it should be interpreted as a summary signal rather than a single precise magnitude of effect. When bruxism activity was instead assessed by questionnaire, clinical global impression, or PSG-scored episode frequency, results were considerably less consistent, with only one of six such assessments showing a clear BoNT-A-specific benefit; this instrumental/subjective divergence, rather than a single unifying effect size, is among the most clinically relevant findings of this review. These EMG-based findings are corroborated, in direction, by three additional studies with non-placebo designs. The treatment shows a favorable short-term safety profile across all eight included studies, with only mild and transient adverse effects reported.

BoNT-A emerges, on the basis of its consistent effect on the mechanistic (EMG-based) outcome evaluated in this review, as a candidate adjunctive therapy for managing moderate to severe SB, particularly in patients unresponsive to conventional measures such as occlusal splints or behavioral interventions, though this review does not provide direct evidence regarding its effect on pain, function, or other bruxism-related symptoms.

Nevertheless, the clinical application of BoNT-A should be approached cautiously. Variability in injection protocols and the absence of long-term safety data highlight the need for further standardization and extended follow-up studies. Additionally, the effectiveness of BoNT-A in awake bruxism (AB) remains uncertain and requires dedicated investigation using objective assessment methods.

In conclusion, BoNT-A represents a mechanistically targeted, adjunctive option within a multidisciplinary management framework for sleep bruxism, on the basis of its consistent effect on masticatory muscle activity, but careful patient selection, precise administration, and ongoing monitoring are essential to maximize benefits and minimize risks.

## Figures and Tables

**Table 1:** PICOS framework defining the review question and eligibility criteria.

Element	Criterion
Population (P)	Adults (≥18 years) diagnosed with sleep bruxism (SB) or awake bruxism (AB), diagnosed as “possible,” “probable,” or “definite” per the graded diagnostic approach (self-report, clinical inspection, and/or instrumental confirmation).
Intervention (I)	Injection of botulinum toxin type A (BoNT-A) into one or more masticatory muscles (masseter and/or temporalis, medial or lateral pterygoid).
Comparison (C)	Placebo (saline) injection, no treatment/untreated arm, or an active comparator (a different BoNT-A injection protocol, muscle combination, or dose).
Outcome (O)	Reduction in bruxism/masticatory muscle activity, measured by EMG amplitude, EMG-derived bruxism events/Bruxism Index, polysomnographically scored rhythmic masticatory muscle activity (RMMA), or validated bruxism-frequency questionnaires/clinical global impression scales, according to whichever method each primary study used. Pain, functional limitation, and quality-of-life outcomes were not evaluated.
Study design (S)	Randomized controlled trials (placebo-controlled or active-comparator) or prospective observational studies; case reports, narrative reviews, editorials, and animal/in vitro studies were excluded.
Timeframe	Studies published between January 2005 and July 2026 (see Section 2.3 for rationale).
Setting/Language	No restriction on clinical setting; articles in English with full text available.

**Table 2:** Summary of the main characteristics of the included studies.

Study	Type of Paper	Treatment Group (n)	Control Group 1 (n)	Control Group 2 (n)	Placebo (n)	Dilution	Total Dose	Type of Botulinum Toxin	Injection Sites	Guidance	Primary Outcome
Cruse et al. (2022) [39]	Double-blind crossover RCT	6	7	9	22	2 mL/100U	/	Type A	Group A: total 60U, 1 site/masseter (30U per masseter). Group B: 1 site/masseter, 3 sites/temporalis; total 90U (30U masseter + 15U temporalis per side). Group C: 1 site/masseter, 3 sites/temporalis, 1 site/medial pterygoid; total 120U (30U masseter + 15U temporalis + 15U medial pterygoid per side).	EMG-guided	Bruxism Index (BI)
Lee et al. (2010) [40]	Parallel-group RCT	6	/	/	6	0.8 mL/80U	80U	Type A	3 sites/masseter	Anatomical landmarks	EMG events
Ondo et al. (2018) [41]	Parallel-group RCT	13	/	/	9	1 mL/100U	100U	Type A	2 sites/masseter (30U per masseter) + 3 sites/temporalis (20U per temporalis)	Anatomical landmarks	EMG bruxing events (masseter, temporalis) – exploratory endpoint
Sherhi et al. (2022) [42]	Parallel-group RCT	11	/	/	11	2 mL/100U	20U	Type A	2 sites/masseter (10U per masseter)	Anatomical landmarks	EMG (masseter, temporalis)
Shim et al. (2014) [38]	Parallel-group RCT	10	10	/	/	4 mL/200U	50U (A)/100U (B)	Type A	Group A: 3 sites/masseter only (25U/muscle, 50U total). Group B: 3 sites/masseter + 3 sites/temporalis (25U/muscle, 100U total).	Anatomical landmarks	EMG peak amplitude
Shim et al. (2020) [43]	Parallel-group RCT	13	/	/	10	2 mL/100U	50U	Type A	2 sites/masseter (25U per muscle)	Anatomical landmarks	EMG peak amplitude
Alzaeem et al. (2025) [44]	Active-comparator parallel RCT	15	15	/	/	1 mL/50U	25U (masseter + LPT) or 15U (masseter only)	Type A	Group 1: 3 sites/masseter (15U) + lateral pterygoid (10U). Group 2: 3 sites/masseter (15U) only.	EMG-guided (LPT), anatomical landmarks (masseter)	EMG (masseter and lateral pterygoid)
Furuhata et al. (2025) [45]	Observational (prospective, single-arm)	304	/	/	/	2.5 mL/100U	25U per side (50U total)	Type A	3 sites/masseter per side (1 at maximal elevation, 2 at base points 1 cm away)	Anatomical landmarks	EMG (masseter amplitude)

Note: Shim et al. (2014) [38] and Alzaeem et al. (2025) [44] compare two active BoNT-A regimens without a placebo arm; Furuhata et al. (2025) [45] is a single-arm observational study without a control group. All three studies are synthesized narratively and were not included in the quantitative pooled analysis (see Section 2 and Section 3).

**Table 3:** Side effects and main results on muscular activity.

Study	Side Effects	*p*-Value (EMG Reduction/ Bruxism Index)	Main Results on Muscular Activity
Cruse et al. (2022) [39]	Mild asymmetry (1 week)	0.003	Group C: largest reduction in BI at 4 weeks (mean BI reduction: −2.15, *p* = 0.009). Groups A and B: smaller, non-significant BI reduction (Group A: *p* = 0.287; Group B: *p* = 0.073). Between-group comparison: no significant differences among treatment groups (F(2,21) = 2.11, *p* = 0.15); no differences between treatment and placebo at 12 weeks. Participants with higher baseline BI showed a greater BTX-A effect after 4 weeks (F(1,21) = 7.76, *p* = 0.012). Subjective scales: no significant differences between active and placebo phases at 4 and 12 weeks, across all BTX-A doses.
Lee et al. (2010) [40]	None	0.006	Within-group comparisons: no significant differences in EMG events at 4, 8, and 12 weeks across any muscle-injection groups (all *p* > 0.20). Between-group comparisons (averaged over 4, 8, and 12 weeks): masseter, botulinum (mean = 0.23 ± 0.29) significantly lower than saline (mean = 2.47 ± 1.23, *p* < 0.001); temporalis, no significant difference between botulinum (mean = 2.22 ± 1.54) and saline (mean = 1.85 ± 1.51, *p* = 0.483). Significant reduction in bruxism events after botulinum toxin injection in the masseter muscle; no significant changes in the temporalis muscle.
Ondo et al. (2018) [41]	Cosmetic change in the smile	0.09	Change in events and total bruxing time: *p* = 0.09 (2-tailed *t*-test; not statistically significant).
Sherhi et al. (2022) [42]	Pain at injection points (1st week); discomfort at injection site	0.013	Mean EMG activity was statistically lower in the BO group compared to the PB group at T1 (*p* = 0.013 and *p* = 0.003 for the right and left masseter, respectively) and T2 (*p* = 0.015 and *p* = 0.008 for the right and left masseter, respectively).
Shim et al. (2014) [38]	None	0.001	BoNT-A reduces the intensity rather than the generation of the contraction in jaw-closing muscles.
Shim et al. (2020) [43]	None	0.0001	The injection decreased the peak amplitude of EMG bursts during SB, only in the treatment group, for 12 weeks.
Alzaeem et al. (2025) [44]	None reported beyond expected transient injection effects	*p* > 0.05 (masseter, between-group); *p* < 0.001 (lateral pterygoid, between-group)	Both groups showed significant within-group reduction in masseter EMG amplitude at 2 weeks (no significant between-group difference); the group also injected in the lateral pterygoid muscle showed significantly greater reduction in lateral pterygoid EMG amplitude.
Furuhata et al. (2025) [45]	No obvious complications observed (n = 304)	*p* < 0.001 (pre-post, masseter EMG amplitude)	Masseter EMG amplitude decreased from 189.0 (SD 120.5) µV to 55.4 (SD 32.9) µV at 2 months; reduction rate was significantly predicted by age, sex, and baseline amplitude (multivariate analysis).

Note: the *p*-values listed in the third column above refer to different statistical contrasts across studies (e.g., between-group comparisons, muscle-specific comparisons, or within-group pre/post comparisons, as detailed for each study in the corresponding cell of the final column) and should not be compared directly with one another across rows.

**Table 4:** Risk of bias assessment (Cochrane Risk of Bias 2.0).

Study	Design	Randomization	Allocation	Blinding	Incomplete Outcome Data	Selective Reporting
Cruse et al. (2022) [39]	RCT	Some concerns	Low	Low	Low	Low
Lee et al. (2010) [40]	RCT	Low	Low	Low	Low	Low
Ondo et al. (2018) [41]	RCT	Low	Low	Low	Low	Low
Sherhi et al. (2022) [42]	RCT	Low	Low	Low	Low	Low
Shim et al. (2014) [38]	RCT	Some concerns	Low	Some concerns	Low	Low
Shim et al. (2020) [43]	RCT	Low	Low	Low	Low	Low
Alzaeem et al. (2025) [44]	RCT	High	Some concerns	Some concerns (unblinded surgeon)	Low	Low

Note: risk-of-bias domains (randomization, allocation, blinding, incomplete data, selective reporting) are shown for all RCTs for completeness. Shim et al. (2014) [38] and Alzaeem et al. (2025) [44] are active-comparator RCTs without a placebo arm and were assessed for internal validity but excluded from the quantitative pooled analysis (see Section 2). For correspondence with the current Cochrane RoB 2 domain structure [34], the domains above map as follows: “Randomization” and “Allocation” together correspond to RoB 2 Domain 1 (bias arising from the randomization process); “Blinding” corresponds jointly to Domain 2 (bias due to deviations from intended interventions, e.g., the unblinded surgeon in Alzaeem et al. [44]) and Domain 4 (bias in measurement of the outcome, e.g., blinded EMG evaluators); “Incomplete Outcome Data” corresponds to Domain 3 (bias due to missing outcome data); and “Selective Reporting” corresponds to Domain 5 (bias in selection of the reported result). The overall risk-of-bias judgment for each study reported above reflects the least favorable rating across these mapped domains; authors should confirm and, if needed, expand this into a full Domain 1–5 table with outcome-specific judgments before final submission; a first version of this expanded, outcome-specific table (Section 3.9) and an explicit GRADE domain-specific rationale (Section 3.10) have now been added below.

**Table 5:** GRADE Summary of Findings for the three outcomes evaluated in this review. Certainty ratings follow standard GRADE symbols: ⊕⊕◯◯ Low, ⊕◯◯◯ Very low. Downgrading rationale for each outcome is detailed in Section 3.10.

Outcome	No. of Participants (Studies)	Summary Statistic	Effect/Findings	Certainty (GRADE)	Reasons for Downgrading
EMG-based muscle activity magnitude (event frequency + amplitude constructs)	101 (5 RCTs)	Hedges’ g = −1.09 (95% CI: −1.65 to −0.54), secondary composite; construct-specific: g = −0.70 (frequency, 3 studies)/g = −1.31 (amplitude, 2 studies), primary	BoNT-A significantly reduces EMG-detectable masticatory muscle activity vs. placebo	⊕⊕◯◯ LOW	Downgraded for risk of bias (−1: majority “some concerns” on RoB 2) and inconsistency (−1: I^2^ = 58.6%; construct heterogeneity)
Questionnaire-, CGI-, and PSG-frequency-based bruxism outcomes	Narratively synthesized across 6 assessments in 5 studies (no pooled N)	Not pooled (narrative synthesis); only 1 of 6 assessments found a clear BoNT-A-specific benefit	No consistent BoNT-A-specific benefit on subjectively/frequency-assessed bruxism	⊕◯◯◯ VERY LOW	Downgraded for risk of bias (−1), inconsistency (−1: 5/6 assessments null), and imprecision (−1: small samples, no pooling possible)
Short-term safety (adverse events)	101 (5 placebo-controlled RCTs)	Mild, transient adverse effects reported in 3/5 RCTs; no serious adverse events in any trial	BoNT-A appears safe over short-term follow-up (2 weeks–6 months)	⊕⊕◯◯ LOW	Downgraded for imprecision (−1: small controlled samples, low event counts) and indirectness (−1: heterogeneous dosing/muscles injected across trials)

## Data Availability

The data supporting the findings of this systematic review are available within the article. The review protocol was registered in PROSPERO (ID: CRD420251037536).

## Supplementary Materials

The following supporting information can be downloaded at: https://www.mdpi.com/article/10.3390/jcm15187179/s1, Supplementary Material S1: Ondo et al. (2018) [41]—Change-Score Sensitivity Analysis; Supplementary Material S2: Cruse et al. (2022) [39]—Crossover Design Sensitivity Analysis; Supplementary Material S3: Impact on the Frequency/Events Subgroup Pooled Estimate; Supplementary Material S4: PRISMA 2020 Checklist.

## Abbreviations

The following abbreviations are used in this manuscript:

AB	Awake bruxism
BI	Bruxism Index
BoNT	Botulinum neurotoxin
BoNT-A	Botulinum toxin type A
CGI	Clinical global impression
CI	Confidence interval
EMG	Electromyography
GRADE	Grading of Recommendations Assessment, Development and Evaluation
PICO	Population, Intervention, Comparison, Outcome
PRISMA	Preferred Reporting Items for Systematic Reviews and Meta-Analyses
PSG	Polysomnography
RCT	Randomized controlled trial
RMMA	Rhythmic masticatory muscle activity
RoB 2	Cochrane Risk of Bias 2.0 tool
SB	Sleep bruxism
SMD	Standardized mean difference
TMD	Temporomandibular disorder
VAS	Visual analog scale
