# Supplementary material for: Effects of Botulinum Toxin Injection on Masticatory Muscle Activity in Sleep Bruxism: A Systematic Review and Meta-Analysis
Source: J Clin Med. 2026 Sep 16;15(18):7179. doi: 10.3390/jcm15187179 (PMC13607196; doi:10.3390/jcm15187179)
Supplement: Supplementary file 1 [file jcm-15-07179-s001.zip › jcm-4550486-supplementary.pdf]

Supplementary Materials

Manuscript: “The Effect of Botulinum Toxin Injection in the Masticatory Muscles on Sleep Bruxism Activity, with Limited Evidence on Awake Bruxism: A Systematic Review”

This file provides the complete calculations and assumptions underlying the correlation-based sensitivity analysis reported in Section 3.4 of the main manuscript, for the two studies (Cruse et al. [39] and Ondo et al. [41]) whose standardized effect sizes required an approximating assumption because neither publication reported a direct group-mean comparison compatible with standard Hedges’ g calculation. All values below were computed directly from the means, standard deviations, and sample sizes reported in the original publications; no data were estimated, interpolated, or otherwise invented.

S1. Ondo et al. (2018) — Change-Score Sensitivity Analysis

Ondo et al. reported bruxing-event frequency (events/hour) only as pre-post means and SDs within each arm, rather than a direct between-group comparison at a single timepoint. The standardized mean difference was therefore derived from the between-group difference in within-arm change scores, which requires an assumption about the correlation (r) between each participant's baseline and follow-up measurement — a value not reported in the original publication.

S1.1 Input data (from Ondo et al., Table/Results)

Arm	n	Baseline mean ± SD	Follow-up mean ± SD	Mean change
BoNT-A	13	9.18 ± 8.48	6.95 ± 7.04	−2.23
Placebo	9	4.63 ± 3.45	10.65 ± 9.57	+6.02

S1.2 Formulas

$$Var(change)\_arm = SD\_pre^2 + SD\_post^2 - 2 \cdot r \cdot SD\_pre \cdot SD\_post$$

$$SD\_pooled = \sqrt{\{ [(n_1-1) \cdot Var(change)_1 + (n_2-1) \cdot Var(change)_2] / (n_1+n_2-2) \}}$$

$$d = (mean\_change\_BoNT{-}A - mean\_change\_placebo) / SD\_pooled = (-2.23 - 6.02) / SD\_pooled = -8.25 / SD\_pooled$$

$$J \text{ (small-sample correction)} = 1 - 3 / [4 \cdot (n_1+n_2-2) - 1]; \quad Hedges' \, g = d \times J$$

$$Var(d) = (n_1+n_2)/(n_1 \cdot n_2) + d^2 / [2 \cdot (n_1+n_2-2)]; \quad SE(g) = J \times \sqrt{Var(d)}$$

With $n_1 = 13$, $n_2 = 9$: $n_1+n_2-2 = 20$, so $J = 1 - 3/79 = 0.9620$, and $(n_1+n_2)/(n_1 \cdot n_2) = 22/117 = 0.1880$.

S1.3 Results across assumed correlations

r	Var(change) BoNT-A	Var(change) Placebo	SD_pooled	d	Hedges' g	SE(g)	95% CI
0.0	121.47	103.49	10.690	−0.772	−0.742	0.433	(−1.591, 0.107)
0.3	85.65	83.68	9.212	−0.896	−0.862	0.439	(−1.722, −0.002)
0.5	61.77	70.47	8.078	−1.021	−0.983	0.445	(−1.855, −0.110)
0.7	37.89	57.26	6.756	−1.221	−1.175	0.457	(−2.070, −0.280)
0.9	14.01	44.06	5.102	−1.617	−1.556	0.484	(−2.505, −0.606)

The main manuscript uses $r = 0$ ($g = -0.74$) as the conservative, pre-specified assumption. As shown above, the magnitude of the standardized effect increases with the assumed correlation, but the direction (favoring BoNT-A) is unchanged across the full range tested; the 95% CI first excludes zero at $r \approx 0.3$.

S2. Cruse et al. (2022) — Crossover Design Sensitivity Analysis

Cruse et al. is a crossover trial reporting a paired active-vs-placebo mean difference in Bruxism Index (BI, events/hour) at 4 weeks (mean = −1.66, n = 22 analyzed), together with the SD of BI in each phase, but not the SD of the within-subject difference itself or the baseline-follow-up correlation. The main manuscript standardizes this mean difference by the baseline SD (a between-subject reference), which does not require a correlation assumption but is a less formally appropriate standardizer for a paired design, as noted by the

reviewer. We therefore additionally computed an alternative, paired-difference standardized effect, which uses the SD of the within-subject difference (itself a function of the assumed correlation r) as the standardizer.

S2.1 Input data (from Cruse et al., Table 2)

Phase	n	Mean ± SD (4 weeks)
Active	22	6.67 ± 4.09
Placebo	22	8.33 ± 4.11
Baseline (both phases)	22	8.29 ± 2.88

Mean difference (active − placebo) = −1.66 (as reported).

S2.2 Method A (main manuscript): baseline-SD standardizer

$$g = \text{mean difference} / \text{baseline SD} = -1.66 / 2.88 = -0.576$$

This point estimate does not depend on r. It was retained as the primary estimate in Section 3.4 of the main manuscript because it was the method used across the review for consistency; it is not perfectly correct for a paired design, hence Method B below.

S2.3 Method B (sensitivity analysis): paired-difference standardizer

$$SD\_diff(r) = \sqrt{(SD\_active^2 + SD\_placebo^2 - 2 \cdot r \cdot SD\_active \cdot SD\_placebo)} = \sqrt{(33.6202 - 33.6198 \cdot r)}$$

$$d\_z(r) = \text{mean difference} / SD\_diff(r) = -1.66 / SD\_diff(r)$$

$$SE(d\_z) \approx \sqrt{(1/n + d\_z^2/(2n))},\ \ n = 22$$

r	SD_diff	d_z (= g, paired)	SE(g)	95% CI
0.0	5.798	−0.286	0.218	(−0.712, 0.140)
0.3	4.851	−0.342	0.219	(−0.772, 0.088)
0.5	4.100	−0.405	0.222	(−0.840, 0.030)
0.7	3.176	−0.523	0.227	(−0.968, −0.077)
0.9	1.834	−0.905	0.253	(−1.401, −0.409)

Under this alternative, more formally appropriate parameterization, the baseline-SD estimate used in the main text (g = −0.58) corresponds to an implied correlation of approximately r ≈ 0.6–0.65 (interpolating between the r = 0.5 and r = 0.7 rows above), i.e., a moderate-to-fairly-high baseline-follow-up correlation, which is plausible for a repeated physiological measure such as EMG-derived bruxism-event frequency in the same individuals but cannot be confirmed without access to the original participant-level data.

S3. Impact on the Frequency/Events Subgroup Pooled Estimate

The frequency/events subgroup (Section 3.4 of the main manuscript) pools Cruse et al., Lee et al. [40], and Ondo et al. [41] using fixed-effect inverse-variance weighting. Lee et al.'s effect size is directly computable from reported group means/SDs and does not require any approximating assumption (g = −2.48, SE = 0.770, computed directly as a standard two-independent-groups Hedges' g); it is held fixed across all rows below. Substituting the Method-B (paired-difference) Cruse estimate and the change-score Ondo estimate at each assumed correlation r yields:

r	Cruse g (SE)	Ondo g (SE)	Lee g (SE) [fixed]	Pooled g	Pooled SE	95% CI
0.0	−0.286 (0.218)	−0.742 (0.433)	−2.483 (0.770)	−0.504	0.189	(−0.874, −0.135)
0.3	−0.342 (0.219)	−0.862 (0.439)	−2.483 (0.770)	−0.570	0.190	(−0.943, −0.198)
0.5	−0.405 (0.222)	−0.983 (0.445)	−2.483 (0.770)	−0.642	0.192	(−1.019, −0.266)
0.7	−0.523 (0.227)	−1.175 (0.457)	−2.483 (0.770)	−0.772	0.197	(−1.157, −0.386)
0.9	−0.905 (0.253)	−1.556 (0.484)	−2.483 (0.770)	−1.157	0.215	(−1.579, −0.735)

Formula used for fixed-effect pooling: $weight\_i = 1/SE\_i^2$; pooled $g = \Sigma(w\_i \cdot g\_i)/\Sigma(w\_i)$; pooled $SE = \sqrt{(1/\Sigma w\_i)}$.

As reported in the main manuscript (Section 3.4), the pooled frequency-subgroup estimate remains negative with a 95% CI excluding zero across the entire tested range of r (0 to 0.9), supporting the qualitative robustness of the conclusion that BoNT-A reduces bruxism-event frequency, while its precise magnitude (−0.50 to −1.16) is sensitive to the unreported baseline-follow-up correlation in the two approximated studies.
