# Supplementary material for: Effects of Botulinum Toxin Injection on Masticatory Muscle Activity in Sleep Bruxism: A Systematic Review and Meta-Analysis
Source: J Clin Med. 2026 Sep 16;15(18):7179. doi: 10.3390/jcm15187179 (PMC13607196; doi:10.3390/jcm15187179)
Supplement: Supplementary file 1 [file jcm-15-07179-s001.zip › jcm-4550486-PRISMA 2020 Checklist.pdf]

S4. PRISMA 2020 Checklist

Manuscript: “Effects of Botulinum Toxin Injection on Masticatory Muscle Activity in Sleep Bruxism: A Systematic Review and Meta-Analysis”

Based on: Page MJ, McKenzie JE, Bossuyt PM, et al. The PRISMA 2020 statement: an updated guideline for reporting systematic reviews. BMJ 2021;372:n71.

The “Location” column below cites the specific manuscript section, table, or figure reporting each item.

Section/Topic	Item #	Checklist Item	Location Where Item Is Reported
Title	1	Identify the report as a systematic review.	Title page (“...A Systematic Review and Meta-Analysis”)
Abstract	2	See the PRISMA 2020 for Abstracts checklist (structured summary of background, methods, results, conclusions).	Abstract (Background/Objectives, Methods, Results, Conclusions)
Introduction	3	Rationale: describe the rationale for the review in the context of existing knowledge.	Introduction, paragraphs 1–3
Introduction	4	Objectives: provide an explicit statement of the objective(s) or question(s) the review addresses.	Introduction, closing paragraph (objective statement)
Methods	5	Eligibility criteria: specify inclusion/exclusion criteria and how studies were grouped for syntheses.	Section 2.3 (Eligibility Criteria)
Methods	6	Information sources: specify all databases/registers/other sources searched, and date each was last searched.	Section 2.4 (Search Strategy): PubMed, Scopus, Web of Science; original search April 2026, PubMed update search July 2026
Methods	7	Search strategy: present full search strategies for all databases, including filters/limits.	Section 2.4: reconstructed Boolean strings for PubMed, Scopus, and Web of Science
Methods	8	Selection process: methods for deciding study eligibility, number/independence of reviewers, automation tools if any.	Section 2.5: three reviewers (M.V., A.C., M.P.) independently screened; fourth reviewer (L.N.) adjudicated discrepancies
Methods	9	Data collection process: methods for collecting data, number/independence of reviewers, automation tools if any.	Section 2.5: data extraction performed independently and in duplicate (M.V., M.P.), third reviewer (A.C.) for disagreements
Methods	10a	Data items: list and define all outcomes sought; how results compatible with each outcome domain were selected.	Section 2.2 (PICO, Outcome definition)
Methods	10b	Data items: list and define all other variables for which data were sought.	Section 2.5 (Data extracted: authors, year, country, design, sample size, bruxism type, dose/site, comparator,

			follow-up, outcome measures, adverse events)
Methods	11	Study risk of bias assessment: methods and tool(s) used, number/independence of assessors.	Section 2.6 (Risk of Bias Assessment: RoB 2 for RCTs, Newcastle–Ottawa Scale for the observational study)
Methods	12	Effect measures: specify the effect measure(s) used for each outcome.	Section 2.7 (Hedges' g standardized mean difference with 95% CI)
Methods	13a	Synthesis: processes for deciding which studies were eligible for each synthesis.	Section 2.7 (five placebo-controlled RCTs eligible for quantitative synthesis; three non-placebo studies synthesized narratively)
Methods	13b	Synthesis: methods to prepare data for synthesis (handling missing statistics, data conversions).	Section 2.7 and Section 3.4 (approximating assumptions for Cruse et al. and Ondo et al., detailed further in Supplementary Materials)
Methods	13c	Synthesis: methods used to tabulate or visually display results of individual studies and syntheses.	Table 3 (individual study results); Figure 2 (forest plot)
Methods	13d	Synthesis: methods used to synthesize results, with rationale.	Section 2.7 (random-effects DerSimonian–Laird model, with rationale; fixed-effect model as sensitivity analysis)
Methods	13e	Synthesis: methods to explore possible causes of heterogeneity (e.g., subgroup analysis, meta-regression).	Section 2.7/3.4 (post hoc construct-based subgroup analysis: event frequency vs. EMG amplitude)
Methods	13f	Synthesis: sensitivity analyses conducted to assess robustness of results.	Section 3.4 (fixed-effect model, leave-one-out analysis, correlation-based sensitivity analysis for Cruse et al. and Ondo et al.; full calculations in Supplementary Materials)
Methods	14	Reporting bias assessment: methods used to assess risk of bias due to missing results.	Section 2.7/3.10: formal tests (funnel plot, Egger's test) not performed given k = 5 studies (below the recommended minimum of 10), stated explicitly
Methods	15	Certainty assessment: methods used to assess certainty in the body of evidence.	Section 2.6 (GRADE approach specified); Section 3.10 (domain-specific GRADE rationale)
Results	16a	Study selection: results of search/selection, from records identified to studies included, ideally with a flow diagram.	Section 3.1; Figure 1 (PRISMA flow diagram)
Results	16b	Study selection: cite studies that might appear eligible but were excluded, and explain why.	Figure 1 gives exclusion counts and reasons by category at each stage; individual excluded-study citations are

			not itemized beyond these counts — authors should confirm whether a full excluded-studies list is required by the Editor
Results	17	Study characteristics: cite each included study and present its characteristics.	Section 3.1; Table 2 (Summary of main characteristics of included studies)
Results	18	Risk of bias in studies: present assessments of risk of bias for each included study.	Table 4 (RoB 2 summary); Section 3.9 (outcome-specific domain-level judgments)
Results	19	Results of individual studies: summary statistics and effect estimates with precision, ideally in tables/plots.	Table 3 (side effects and main results); Figure 2 (forest plot with individual and pooled effect sizes and 95% CIs)
Results	20a	Synthesis: briefly summarize characteristics and risk of bias among studies contributing to each synthesis.	Section 3.4, opening paragraphs; cross-referenced to Table 4
Results	20b	Synthesis: present results of all statistical syntheses, including summary estimates, precision, and heterogeneity.	Section 3.4 (pooled Hedges' g, 95% CI, I^2, Cochran's Q for the overall and construct-specific estimates)
Results	20c	Synthesis: present results of investigations of possible causes of heterogeneity.	Section 3.4 (construct-based subgroup analysis: frequency vs. amplitude, with between-subgroup test)
Results	20d	Synthesis: present results of all sensitivity analyses.	Section 3.4 (fixed-effect model, leave-one-out analysis, correlation-based sensitivity analysis); Supplementary Materials (full calculations)
Results	21	Reporting biases: present assessments of risk of bias due to missing results for each synthesis.	Section 3.10 (publication bias not formally testable given $k = 5$; stated explicitly rather than assessed graphically)
Results	22	Certainty of evidence: present assessments of certainty for each outcome.	Section 3.10 and Table 5 (GRADE Summary of Findings table)
Discussion	23a	Provide a general interpretation of results in the context of other evidence.	Section 4 (Discussion, opening and mechanism paragraphs)
Discussion	23b	Discuss limitations of the evidence included in the review.	Section 4.2 (Limitations, points 1–5)
Discussion	23c	Discuss limitations of the review processes used.	Section 4.2 (Limitations, points on search exhaustiveness and approximated effect sizes)
Discussion	23d	Discuss implications of the results for practice, policy, and future research.	Section 4.1 (Clinical Implications) and Section 4.3 (Future Directions)
Other information	24a	Provide registration information, including register name and registration number, or state the review was not registered.	Section 2.1: registered on PROSPERO (ID: CRD420251037536)

Other information	24b	Indicate where the review protocol can be accessed, or state a protocol was not prepared.	Section 2.1 references the PROSPERO registration;
Other information	24c	Describe and explain any amendments to information provided at registration or in the protocol.	Not currently reported in the manuscript
Other information	25	Describe sources of financial or non-financial support, and the role of funders/sponsors.	Funding statement: “This research received no external funding.”
Other information	26	Declare any competing interests of review authors.	Conflicts of Interest statement: “The authors declare no conflicts of interest.”
Other information	27	Report which of the following are publicly available: data collection forms, extracted data, analysis data, analytic code, other materials.	Data Availability Statement (data within the article); Supplementary Materials (full sensitivity-analysis calculations/code description)
